# Mortality reduction in pediatric patients with severe fatal human adenoviral pneumonia treated with high titer neutralizing antibodies (NAbs) plasma: a retrospective cohort study

**DOI:** 10.1186/s12887-022-03225-1

**Published:** 2022-03-22

**Authors:** Hongyan Peng, Feiyan Chen, Yunlong Zuo, Bolun Huang, Yiyu Yang, Run Dang

**Affiliations:** Department of Pediatric Intensive Care Unit, Guangzhou Women and Children’s Medical Center, No.318 Renmin Middle Road, Yuexiu District, Guangzhou, 510120 China

**Keywords:** Human adenovirus (HAdV), Neutralizing antibodies (NAbs), Pediatric, Pneumonia, Mortality

## Abstract

**Background:**

Severe fatal human adenoviral (HAdV) pneumonia is associated with significant mortality and no effective drug is available for clinical therapy. We evaluated the association and safety of high titer neutralizing antibodies (NAbs) plasma in pediatric patients with severe fatal HAdV pneumonia.

**Methods:**

A retrospective cohort study was performed between January 2016 to June 2021 in pediatric intensive care unit. Pediatric patients with severe fatal HAdV pneumonia were included and divided into plasma group (conventional treatment plus high titer NAbs plasma treatment) and control group (conventional treatment alone). The primary outcome was mortality in hospital. Secondary outcomes were the duration of fever after adenovirus genotype determined, duration of invasive mechanical ventilation, length of hospital stay. T-test, Mann-Whitney U-test, chi-square test, univariable and multivariable logistic regression analysis, Kaplan-Meier method and log-rank test were adopted to compare differences between two groups.

**Results:**

A total of 59 pediatric patients with severe fatal HAdV pneumonia were enrolled. They were divided into plasma group (*n* = 33) and control group (*n* = 26). The mortality in hospital was 28.8% (17/ 59). Significantly fewer patients progressed to death in plasma group than control group (18.2% vs 42.3%, *p* = 0.042). Sequential organ failure assessment (SOFA) score, oxygen index (OI) and high titer NAbs plasma treatment were included in multivariable logistic regression analysis for mortality risk factors. Consequentially, SOFA score (Hazard Ratio [HR] 7.686, 95% Confidence Interval [CI] 1.735–34.054, *p* = 0.007) and without high titer NAbs plasma treatment (HR 4.298, 95%CI 1.030–17.934, *p* = 0.045) were significantly associated with mortality. In addition, high titer NAbs plasma treatment were associated with faster temperature recovering in survivors (*p* = 0.031). No serious adverse effects occurred.

**Conclusions:**

Administration of high titer NAbs plasma were associated with a lower hazard for mortality in pediatric patients with severe fatal HAdV pneumonia. For survivors, high titer NAbs plasma treatment shorten the duration of fever.

## Background

Human adenovirus (HAdV) infection is a common respiratory tract pathogen. In severe acute respiratory infection, the prevalence of HAdV has been reported to 20.1% in Northern China and 8.2% in Eastern China [1]. 88.2% were pediatric patients in severe acute respiratory infection patients [2]. The mortality rate of immunodeficiency patients was 136 times higher than that of patients with normal immune function [3]. Adenovirus antibody levels increase with age. At present, HAdV-3 and HAdV-7 are the most important types in pediatric patients with severe respiratory diseases. In a study of adenovirus antibody levels in Chinese children found that the seropositive rates were increased against HAdV-3 (12.07, 33.96, 64.29%) and against HAdV-7 (0, 18.87, 19.05%) in different age groups (1–2, 3–5, 6–17 years, respectively) [4]. The severity of HAdV infection in respiratory tract is various from mild upper respiratory symptoms to severe fatal HAdV pneumonia. Adenovirus pneumonia was severe in 20% of patients, with a fatality rate of 5.4% [5]. Furthermore, the survivors had long term sequelae following severe HAdV pneumonia include recurrent wheeze (5.4%), bronchiolitis obliterans (15.2%) [5] and bronchiectasis (50%) [6]. However, there is no specific and effective treatment for severe fatal HAdV pneumonia.

Based on the assumption that immune plasma can establish passive immunization to control the progression of the disease before the infected patient develop a specific immunization. For almost a century, convalescent plasma and the products of convalescent blood such as specific immunoglobulin have been developed to treat virus infection disease when no specific therapy was available [7, 8]. Especially in recent years, convalescent plasma transfusion was observed in patients infected with influenza virus, middle east respiratory syndrome coronavirus (MERS-CoV),severe acute respiratory syndrome, ebola virus, and coronavirus COVID-19 [9–12]. However, these treatments are difficult to obtain or expensive to produce, and may not be able to control the progression of infection disease in emergency situations. Therefore, we suggest that high titer neutralizing antibodies (NAbs) plasma screened from healthy blood donors may be beneficial for the control of severe fatal HAdV pneumonia. To evaluate clinical curative effect and safety of high titer NAbs plasma treatment, we conducted a retrospective cohort study in our hospital for a 5-year period in pediatric patients with severe fatal HAdV pneumonia.

## Methods

### Study design and population

A retrospective cohort study was performed in patients with severe fatal HAdV pneumonia at Guangzhou Women and Children’s Medical Center between January 2016 to June 2021. This study was approved by the ethics committee of Guangzhou Women and Children’s Medical Center (Approval number: 2016102420). Signed informed consents were obtained from pediatric parents or guardians for high titer NAbs plasma treatment. Inclusion criteria were as follows:(1) age below 18 years; (2) adenoviral infection diagnosis based on polymerase chain reaction (PCR) tests by using nasopharyngeal swab, sputum or bronchoalveolar lavage. Adenovirus PCR detection kit was provided by Guangdong Hexin Health Technology Company. We carried out routine adenovirus detection for hospitalized children with severe pneumonia and monitored them by qPCR method. However, adenovirus genotype was not performed in this first test, and a relatively conservative and universal test containing 300 bp target gene fragment was used. We genotyped adenovirus-positive patients by qPCR method, and differentiated them according to the different gene fragments of different adenovirus-typing. Because adenovirus genotyping technology was not mature at that time, we only tested several common adenovirus genotypes, (genotypes 3, 4, 7, 11 and 55). However, genotype 3 and 7 were mainly concentrated in our included patients, 2 patients did not belong to any of the above genotypes, so they were classified as unknown type, included in the control group and all survived. (3) pneumonia confirmed by the presence of parenchymal infiltration on chest imaging with respiratory symptoms; (4) In order to make the pediatric patients had comparability, we selected patients with critically ill adenovirus pneumonia. We defined it as severe fatal adenovirus pneumonia. Consistented with severe fatal: acute respiratory distress syndrome [13], performed invasive mechanical ventilation and Murray score [14] was greater than two. Exclusion criteria were as follows: (1) immunoglobulin or plasma allergy; (2) patients with serious underlying diseases (such as chronic lung disease, complex congenital heart disease, severe liver or kidney disease, malignant tumors, severe malnutrition, severe immunodeficiency, genetic metabolic diseases, brain dysfunction due to various causes prior to onset.).

### Clinical and laboratory data

The information of each patient was collected from electronic medical records. Information as follows: (1) basic information: age, weight, sex, underlying comorbidity and date of admission; (2) clinical features before adenovirus genotype determined: fever days, pathogen, laboratory indicators of infection and organ function, sequential organ failure assessment (SOFA) score, oxygen index (OI). (SOFA score were grouped at the boundary of 7 based on the median SOFA score in the included patients. The median OI and quartile at the time of enrollment were 9.74(6.44–17.99), and the dividing line of mild, moderate and severe ARDS in children were 8 and 16 according to consensus recommendations from the Pediatric Acute Lung Injury Consensus Conference [13] .OI were grouped at the boundary of 8 based on classification limits of mild to moderate lung injury). (3) main management throughout the course of the disease: human immunoglobulin, glucocorticoid, vasoactive drugs and high titer NAbs plasma; (4) primary endpoint: mortality in hospital; (5) secondary clinical outcomes: fever days after adenovirus genotype determined, duration of invasive mechanical ventilation, hospital length of stay. Due to the high mortality rate of the control group and in consideration of data integrity, we only compared secondary clinical outcomes in survivors.

### Protocol of high titer plasma treatment

High titer NAbs plasma were screened from fresh frozen plasma of healthy blood donors. High titer NAbs plasma were classified and stored in the blood center, and all of them were managed according to blood products regulations. The proper high titer NAbs plasma in this study as follows: (1) the level of neutralizing antibodies was above 1:1000 (So far, there is no standard definition of high titer NAbs plasma. We retained adenovirus NAbs plasma with titer > 1:1000 through Guangzhou Blood Center screening); (2) the ABO blood type was compatible with patients’ ABO blood type; (3) the NAbs against HAdV type (in this study we mainly include HAdV-3 and HAdV-7) were compatible with patients’ HAdV type. High titer NAbs plasma regimens were started within 24 h following adenovirus genotype determined. Firstly, we confirmed whether the blood center had the proper high titer NAbs plasma. Secondly, we signed the informed consent form with patient’s parents or guardians. In the study, no patient withdrew informed consent form before receiving the intervention. Finally, the volume, frequency and infusion speed of high titer NAbs plasma were adjusted according to the patient’s tolerance. On the whole, the transfusion dose of high titer NAbs plasma was approximately 10–20 ml/kg with 2–4 h under supervision of the treating physician. No high titer NAbs plasma would be given if the proper high titer NAbs plasma was not available. The conventional treatments include management with glucocorticoid, human immunoglobulin, other symptom control and supportive treatment [15].

### Statistical analysis

Continuous variables were described as the mean and standard deviation (SD) for normally distributed variables, the median and interquartile range (IQR) for non-normally distributed variables. Categorical variables were presented as number (percentage). The independent sample T-test, Mann-Whitney U-test, chi-square test and Fisher’s exact test were adopted to compare differences between two groups. At the same time, variables with *p*-value were less than 0.1 in univariable analysis were selected for multivariable logistic regression analysis for mortality risk factors. Time-to-event data were analyzed by using the Kaplan-Meier method and log-rank test were used for comparing in survivors. Tests were two-tailed and *p*-value< 0.05 were defined as statistically significant. All statistical analyses were performed with SPSS statistical software, version 26.0 (IBM, USA).

## Results

### Characteristics of the study population

**Fig. 1:**
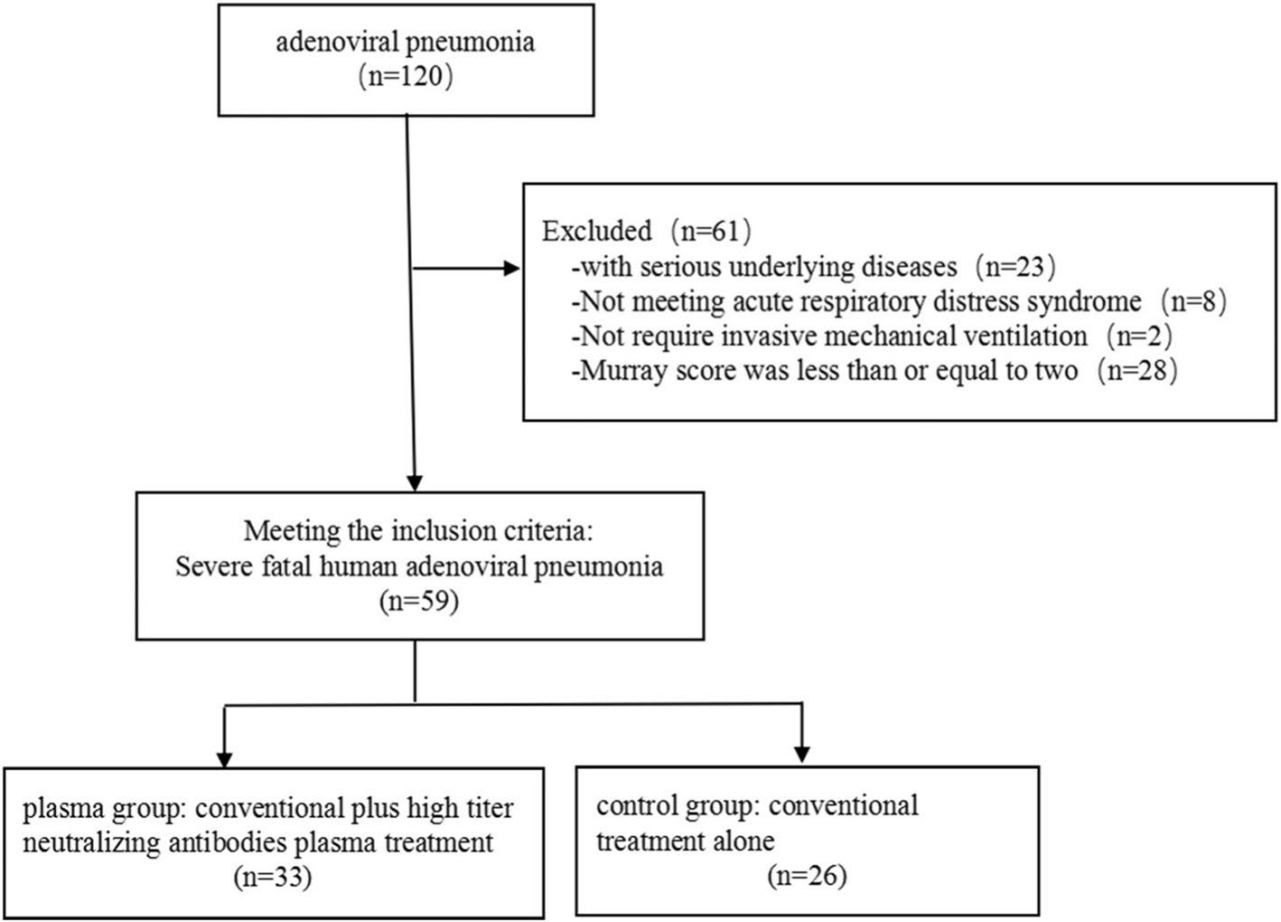
Patient enrolment and study profile

Of the 120 pediatric patients diagnosed with adenoviral pneumonia in PICU between January 2016 to June 2021. Of these, 61 patients were excluded from the study. Consequently, 59 patients were included in the study, 33 patients got conventional plus high titer NAbs plasma treatment (plasma group) and 26 patients got conventional treatment alone (control group). (Fig. 1). Baseline characteristics of these pediatric patients were illustrated in Table 1. In the study population, there were 52 (88.1%) patients with HAdV-7, 5 (8.5%) patients with HAdV-3, and 2 (3.4%) patients with unclear adenovirus genotype. The median age was 18 months (IQR, 10 to 36) and 67.8% were men. The median fever days were 10 days (IQR, 7 to 14) and 28.8% of patients were co-infection before adenovirus genotype determined. More than half of the patients had SOFA score ≥ 7and OI ≥ 8. The final mortality in hospital was 28.8% (17 of 59 patients).

**Table 1:** Characteristics of the all patients, and comparison between plasma group and control group

Variables	Total ***N*** = 59	Plasma group ***N*** = 33	Control group ***N*** = 26	***p*** value
Age (months)	18.00 (10.00–36.00)	18.00 (11.00–38.50)	16.50 (7.75–32.50)	0.647
Male,n (%)	40 (67.8)	23 (69.7)	17 (65.4)	0.725
Weight(kg)	10.00 (9.00–13.00)	10.00 (9.75–13.00)	10.00 (8.28–12.50)	0.386
Fever days^a^	10.00 (7.00–14.00)	11.00 (8.00–14.50)	9.50 (7.00–14.50)	0.540
Co-infection, n (%)	17 (28.8)	12 (36.4)	5 (19.2)	0.149
**Blood routine and infection indicators**				
Leukocyte,10^9^/L	5.60 (2.70–9.70)	5.60 (2.70–8.85)	5.85 (2.60–11.50)	0.867
Hemoglobin,(g/L)	92.00 ± 17.37	88.82 ± 14.79	96.04 ± 19.75	0.114
Neutrophil, 10^9^/L	3.74 (1.48–6.85)	3.74 (1.56–6.84)	1.36 (1.36–8.76)	0.963
Platelets, 10^9^/L	201.00 (117.00–319.00)	188.00 (125.00–254.50)	252.00 (100.00–354.75)	0.296
C reactive protein, mg/L	36.70 (11.88–58.20)	42.10 (12.54–70.35)	27.31 (9.00–54.08)	0.445
**Organ function indicators, n (%)**				
Alanine transaminase>50 U/L	16 (27.1)	10 (30.3)	6 (23.1)	0.535
Serum creatinine >62 umol/L	1 (1.7)	1 (3.0)	0 (0.0)	1.00
Creatine kinase isoenzyme>37 U/L	21 (35.6)	10 (30.3)	11 (42.3)	0.339
Prothrombin time>15 s	11 (18.6)	5 (15.2)	6 (23.1)	0.438
Activated partial prothrombin time>45 s	28 (47.5)	17 (51.5)	11 (42.3)	0.482
Fibrinogen<2 g/L	15 (25.4)	6 (18.2)	9 (34.6)	0.150
**Adenovirus genotype, n (%)**				0.156
ADV3	5 (8.5)	4 (12.1)	1 (3.8)	
ADV7	52 (88.1)	29 (87.9)	23 (88.5)	
Unkown	2 (3.4)	0 (0.0)	2 (7.7)	
**Severity of Illness, n (%)**				
SOFA score ≥ 7	33 (55.9)	15 (42.4)	18 (69.2)	0.068
OI ≥ 8	35 (59.3)	21 (63.6)	14 (53.8)	0.447
**Treatment, n (%)**				
Human immunoglobulin	52 (88.1)	31 (93.9)	21 (80.8)	0.120
Glucocorticoid	35 (59.3)	20 (60.6)	15 (57.7)	0.821
Vasoactive drug	36 (61.0)	20 (60.6)	16 (61.5)	0.942
**Secondary outcomes, (days)**				
Fever days after adenovirus genotype determined	3.00 (2.00–6.00)	3.00 (2.00–5.00)	4.50 (2.00–7.00)	0.232
Duration of invasive mechanical ventilation	14.00 (8.00–22.00)	15.00 (10.00–23.00)	11.00 (7.00–20.00)	0.207
Hospital length of stay	32.00 (21.00–40.00)	17.00 (12.50–25.00)	15.50 (8.50–24.25)	0.249
**Primary outcome**				
Mortality, n (%)	17 (28.8)	6 (18.2)	11 (42.3)	0.042

*SOFA* Sequential organ failure assessment, *OI* Oxygen index, *ADV* Adenovirus

Fever days^a^: Fever days before adenovirus genotype determined

### Comparison between plasma group and control group

There were no significant differences in age, gender, body weight and adenovirus genotype between plasma group and control group (*p*>0.05). Before adenovirus genotype was determined, there were no statistical differences in fever duration, co-infection, SOFA score, OI, blood routine, infection indicators, blood coagulation function, and other organ function indicators (include alanine transaminase,serum creatinine,creatine kinase isoenzyme) (*p* > 0.05). During the entire treatment, there were also no significant differences in glucocorticoid treatment, human immunoglobulin treatment (*p* > 0.05). There were no statistically significant differences in secondary outcomes between the two groups: fever days after adenovirus genotype determined, duration of invasive mechanical ventilation, duration of hospitalization (*p* > 0.05). However, plasma group had lower mortality than control group (18.2% versus 42.3%, *p* = 0.042). As shown in Table 1.

### Comparison between survivors and non-survivors

The clinical characteristics of survivors and non-survivors were shown in Table 2.

**Table 2:** Characteristics between survivors and non-survivors

Variables	Survivors ***N*** = 42	Non-survivors ***N*** = 17	***p*** value
Age (months)	17.5 (7.75–30.00)	21.00 (10.00–52.50)	0.344
Male, n (%)	28 (66.7)	12 (70.6)	0.770
Weight (kg)	10.00 (9.38–12.50)	11.00 (9.00–17.50)	0.400
Fever days^a^	9.50 (7.00–14.25)	11.00 (8.00–15.50)	0.563
Co-infection, n (%)	13 (31.0)	4 (23.5)	0.408
**Blood routine and infection indicators**			
Leukocyte,10^9^/L	5.55 (2.70–9.23)	6.30 (2.60–11.50)	0.960
Hemoglobin,(g/L)	92.26 ± 16.62	91.35 ± 19.63	0.857
Neutrophil, 10^9^/L	3.91 (1.56–6.78)	3.00 (1.11–10.15)	0.867
Platelets, 10^9^/L	195.50 (132.75–302.25)	242.00 (67.50–356.00)	0.947
C reactive protein, mg/L	37.40 (11.66–73.35)	27.52 (9.44–55.00)	0.808
**Organ function indicators, n (%)**			
Alanine transaminase>50 U/L	9 (21.4)	7 (41.2)	0.122
Creatinin>62 umol/L	0 (0.0)	1 (5.9)	0.288
Creatine kinase isoenzyme>37 U/L	13 (31.0)	8 (47.1)	0.242
Prothrombin time>15 s	6 (14.3)	5 (29.4)	0.162
Activated partial prothrombin time>45 s	18 (42.9)	10 (58.8)	0.266
Fibrinogen<2 g/L	9 (21.4)	6 (35.3)	0.216
**Severity of Illness, n (%)**			
SOFA score ≥ 7	18 (42.9)	15 (88.2)	0.001
OI ≥ 8	22 (52.4)	13 (76.5)	0.088
**Treatment, n (%)**			
Human immunoglobulin	38 (90.5)	14 (82.4)	0.320
Glucocorticoid	27 (64.3)	8 (47.1)	0.222
Vasoactive drug	23 (54.8)	13 (76.5)	0.122
High titer NAbs plasma	27 (64.3)	6 (35.3)	0.042
**Adenovirus genotype, n (%)**			
ADV3	4 (9.5)	1 (5.9)	0.578
ADV7	36 (85.7)	16 (94.1)	
Unkown	2 (4.8)	0 (0)	

*SOFA* Sequential organ failure assessment, *OI* Oxygen index, *ADV* Adenovirus, *NAbs* Neutralizing antibodies

Fever days^a^: Fever days before adenovirus genotype determined

Survivors had more patients received high titer NAbs plasma treatment than non-survivors (64.3% versus 35.3%, *p* = 0.042). The rate of SOFA score ≥ 7 was higher in non-survivors (42.9% versus 88.2%, *p* = 0.001). The rate of OI ≥ 8 was 52.4% and 76.5% in survivors and non-survivors(*p* = 0.088). There were no other significant differences in survivors and non-survivors.

### Risk factors for mortality

In order to study risk factors for mortality in patients with severe fatal HAdV pneumonia. Variables with *p*-value were less than 0.1 in univariable analysis were selected for multivariable logistic regression. Consequentially, SOFA score, OI and high titer NAbs plasma treatment were included. When SOFA score and OI were adjusted, high titer NAbs plasma treatment was still a protective factor for patients (HR = 4.298, 95%CI 1.030–17.934, *p* = 0.045). As shown in Table 3.

**Table 3:** Risk factors for mortality in patients with severe fatal human adenoviral pneumonia

Variables	Univariable analysis			Multivariable analysis		
	OR	95%CI	*P*	OR	95%CI	*P*
SOFA score (.<7 versus ≥7)	8.400	2.076–33.982	0.003	7.686	1.735–34.054	0.007
OI (.<8 versus ≥8)	2.955	0.827–10.561	0.096	3.721	0.789–17.550	0.097
High titer NAbs plasma treatment (yes versus no)	3.300	1.016–10.719	0.047	4.298	1.030–17.934	0.045

### Subgroup analysis in survivors

**Fig. 2:**
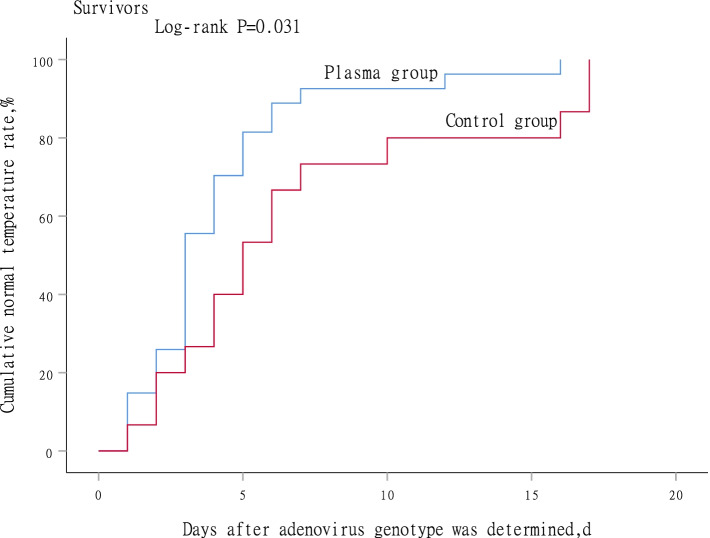
Time to temperature returns to normal between plasma group and control group in survivors

**Fig. 3:**
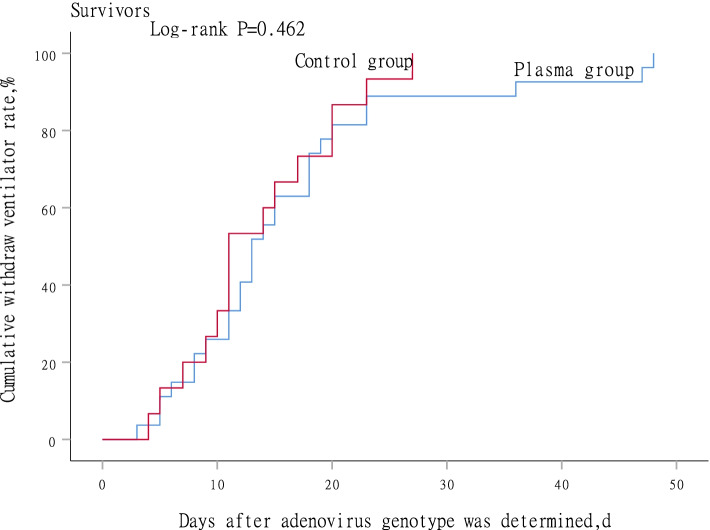
Time to withdraw ventilator between plasma group and control group in survivors

**Fig. 4:**
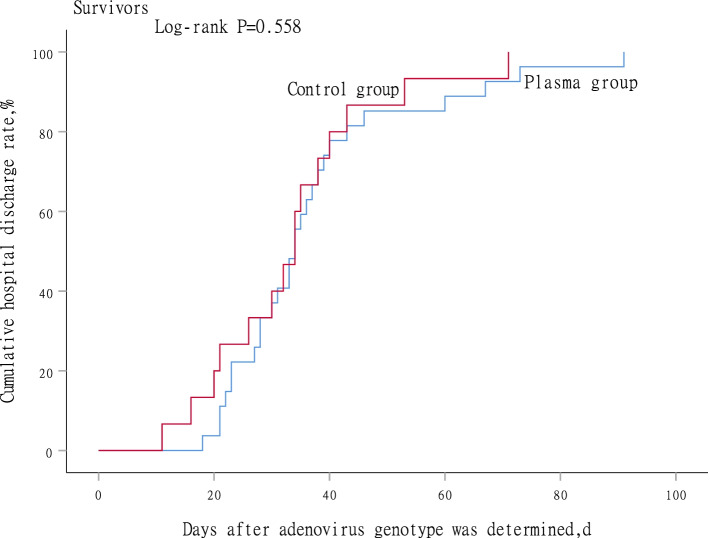
Time to hospital discharge between plasma group and control group in survivors

Subgroup analysis was performed with Kaplan-Meier method and log-rank test to compare differences between plasma group and control group in survivors. High titer NAbs plasma treatment was observed in faster temperature recovering to normal, but not the duration of invasive mechanical ventilation and hospitalization days. As shown in Figs. 2, 3 and 4.

### Adverse events

Only one patient was reported sporadic red rash throughout the body during high titer NAbs plasma transfusion. However, the patient recovered completely soon after intravenous promethazine and a slower rate of plasma infusion. No other adverse reactions were observed in the pediatric patients during high titer NAbs plasma transfusion.

## Discussion

In our study, the administration of high titer NAbs plasma was associated with a lower hazard for mortality in pediatric patients with severe fatal HAdV pneumonia, when SOFA score and OI were adjusted. In addition, further analysis showed that the time to normal body temperature was shortened with high titer NAbs plasma therapy in survivors. However, the duration of mechanical ventilation and length of hospital stay were no significant differences in survivors.

In recent years, there have been increasing reports about NAbs from convalescent patients’ plasma in the treatment of virus-associated pneumonia. A randomized and multicenter phase 2 clinical trial in the United States supported the treatment of NAbs plasma in severe influenza, the mortality of anti-influenza plasma plus standard care and standard care alone were 2% and 10%, respectively [16]. Another non-randomized prospective study also shown that convalescent plasma treatment can reduce 30-day mortality in critically ill patients with COVID-19 disease [17]. The results of these observational studies supported a probable efficacy and safety of NAbs were similar to ours. Conversely, a comparative study in Guinea found the administration of NAbs from convalescent patients could not improve survival in 84 patients with Ebola virus disease, the mortality was 31% in the convalescent plasma treatment group versus 38% in the control group. However, the level of NAbs was unclear in the study, which may affect the effectiveness of treatment [18]. A recent small sample study shown that convalescent plasma therapy contributed to reduce viral load and longer survival in patients with COVID-19. However, convalescent plasma did not reduce the mortality rate in critically ill patients with end-stage COVID-19 [19].

Different criteria of inclusion and exclusion for the study population, levels of NAbs may lead to different therapeutic effects of NAbs plasma. In the current study, the total mortality of 28.8% (17 of 59 patients) was higher than 15%(11 of 75 patients) described in the other observational study [20]. It may indicate that our study population was more serious. Furthermore,the proper high titer NAbs plasma in our study met the following conditions: plasma were screened from fresh frozen plasma with the level of NAbs above 1:1000; ABO blood type were compatible with patients’ ABO blood type; NAbs against HAdV type were compatible with patients’ HAdV type. To our knowledge few investigators had treated HAdV pneumonia with high titer NAbs plasma from healthy blood donors. In a word, our NAbs plasma had high titers and specific to HAdV type. In a retrospective cohort study found high antibody levels of convalescent plasma administration reduced risk of death compared with low antibody levels of convalescent plasma administration [21]. Another study found viral load reduced in the 2 participants who received higher levels of NAbs plasma but not in the 2 participants who received lower levels of NAbs plasma [22]. These studies showed that higher levels of NAbs plasma were beneficial to improve clinical outcomes.

Another reason for different therapeutic effect of NAbs plasma is the application time. However, the entire mechanisms and precise therapeutic components of NAbs plasma are not fully understood. The possible mechanisms of NAbs plasma in virus pneumonia, including directly neutralizing the virus and regulating immune [23]. Therefore, the transfusion of NAbs plasma may be beneficial to control the severe inflammatory response induced by virus and reduce the viral load. In addition, animal and cellular research also had shown that plasma could improve pulmonary vascular permeability, reduce leukocyte infiltration and alveolar wall thickening [24]. Typically, viremia peaks are in the first week after viral infection, and patients commonly trigger primary immune to clear viruses within 2 weeks [25]. Two weeks later, clinical deterioration is the result of inflammatory or hyperimmune attacks rather than viruses directly damage tissue. Therefore, high titer NAbs plasma should theoretically be more effective in the early course of disease. Based on these theories, convalescent plasma transfusion was failure to reduce mortality in some studies might be attributed to the late application of convalescent plasma (on median day 21.5 after onset) [26]. In contrast, one critically ill patient finally recovered after convalescent plasma infused on day 11 after onset [26]. In our study, the median time to give high titer NAbs plasma was on day 10 after onset. Therefore, we observed a reduction in mortality with high titer NAbs plasma therapy.

It is worth mentioning that high titer NAbs plasma belongs to blood products. Transfusion-related adverse events may occur in the transfusion of blood products, including fever (58.5%), anaphylaxis (1.4%), hypotension (0.9%), haemolytic reactions (5.2%), transfusion related acute lung injury (TRALI) (0.3%), transfusion associated circulatory overload (0.6%) and so on [27]. Among them, TRALI is the leading cause of transfusion-related death. Another study had shown that the incidence of TRALI was 5.1% (114 of 2235) [28]. Patients with severe viral infection have significant acute lung injury, plasma transfusion may aggravate lung injury. In our study, most patients tolerated high titer NAbs plasma transfusion well, only 1 patient had scattered red rash throughout the body during the process of high titer NAbs plasma infusion. However, the rash quickly subsided after the treatment of promethazine and a slower rate of plasma infusion, and no other adverse reactions were observed. As everyone knows, Human leukocyte antigen antibodies (HLA-Ab) from the donor are a primary cause of TRALI [29]. Nevertheless, infectious pathogens can trigger the formation and rise of specific HLA-Ab reactivity via multiple mechanisms [30]. Recently, one study performed universal screening of HLA-Ab for all convalescent plasma. They found that 5 (7.2%) of 69 male patients were HLA-Ab positive [31]. Unlike convalescent plasma donors who have recently recovered from viral infection high titer NAbs plasma from regular blood donors may contain little HLA-Ab. As a result, high titer NAbs plasma is relatively safe, but it is still recommended to monitor and manage the possible adverse reactions according to the guidelines related to blood transfusion.

Several limitations of our study should also be recognized. First, this study was conducted in a single center. It could also indicate that the conventional treatment was more consistent. Second, because the proper high titer NAbs plasma was limited, the study was not randomized and the sample size was small. The efficacy of the study was inadequate to estimate the association between treatment and mortality with sufficient adjustment for confounding. A randomized controlled multicenter study will be required in the future. In addition, we did not dynamically monitor the viral load in patients. Viremia peaks in patients with severe fatal HAdV pneumonia may not be consistent, the dynamic monitoring of gland viral load may help to find the best time to use high titer NAbs plasma and clarify mechanism.

## Conclusion

In conclusion, our study may be the first to use high titer NAbs plasma in the treatment of pediatric patients with severe fatal HAdV pneumonia. Furthermore, we found that high titer NAbs plasma therapy may be associated with reduced mortality. However, large-scale studies are required to investigate the optimal time for high titer NAbs plasma transfusion for preventing clinical deterioration and reducing mortality.

## Data Availability

The datasets used and/or analyzed are not publically available in the current study. However, the datasets can be shared from the corresponding author on reasonable request.

## Abbreviations

NAbs: Neutralizing antibodies
HAdV: Human adenoviral
SOFA: Sequential organ failure assessment
OI: Oxygen index
HR: Hazard ratio
CI: Confidence Interval
MERS-CoV: Middle east respiratory syndrome coronavirus
PICU: Pediatric intensive care unit
PCR: Polymerase chain reaction
SD: Standard deviation
IQR: Interquartile range
TRALI: Transfusion related acute lung injury
HLA-Ab: Human leukocyte antigen antibodies
